# Fear of Falling Correlates with Subtle Neuromuscular Balance and Strength Deficits of Fragility Fracture Patients

**DOI:** 10.14336/AD.2016.0302

**Published:** 2016-10-01

**Authors:** Corinne E. Wee, Tyler D. Ames, Khoi M. Le, Tiffany Wang, Laura S. Phieffer, Carmen E. Quatman

**Affiliations:** ^1^Department of Orthopaedics, and; ^2^The Ohio State University College of Medicine, Columbus, OH 43210, USA

**Keywords:** clinical assessment tool, Nintendo® Wii Balance Board, Level Belt, advanced practice provider, fragility fracture

## Abstract

Fragility fractures, or fractures occurring from a low-trauma event, are extremely prevalent among the elderly population worldwide and associated with significant mortality and morbidity. This study evaluated the relationship between FES-I Fear of Falling Survey results, self-reported activity restrictions via the SF-36 survey, and scores recorded by portable, inexpensive clinical assessment tools (CATs) during dynamic functional tasks. Low scores during these tasks may indicate functional deficits that put patients at risk for falls and subsequent fragility fractures. Forty-one subjects (20 fragility fracture patients, 21 controls without history of fragility fractures) over the age of 50 were recruited from three outpatient orthopaedic clinics. All subjects were administered a FES-I Fear of Falling Survey, a portion of an SF-36 survey, and tested using three different portable CATs: the Wii Balance Board, iPod Level Belt and Saehan Squeeze Hand Grip Dynamometer. There were several measured variables that showed a moderate correlation with Fear of Falling scores. Of note, correlations between FES-I scores and maximum hand grip strength for both the dominant hand (R= -0.302, p=0.069) and non-dominant hand (R= -0.309, p=0.059), as well as maximum anterior-posterior sway measured by the iPod Level Belt (R=0.320, p=0.056) were found to be marginally significant. In addition, the correlation between FES-I and average anterior-posterior sway was found to be significant (R=0.416, p=0.012). The Nintendo Wii and iPod Level Belt are relatively inexpensive, portable tools that can assess patients for subtle deficits during dynamic functional tasks. The results indicate that these tools can provide a more objective measure of a patient’s limitations during daily activities such as walking by assigning them a numerical value and correlating this value to physical deficits that impact balance and coordination. In the future, CATs may also have a role in predicting outcomes and in individualizing care, therapy, and at-home preventive measures.

More than 1.5 million fragility fractures occur in the United States each year, leading to reduced quality of life and significant financial burden[1, 2] for the patients who sustain these injuries. Fragility fractures are defined as a fracture occurring from a low-trauma event, such as falling from a standing height. As fragility fractures are so prevalent in the older population, low-cost, effective prevention of even a small percentage of fragility fractures would be economically and medically beneficial.

Current physical exam techniques used to assess musculoskeletal pathology in the clinical setting can detect gross strength and physical abnormalities but often overlook subtle changes in balance, coordination and strength during functional activities that often accompany musculoskeletal pathology. Different balance and strength scales are often employed in clinical settings to provide clinicians with a numerical value of severity, but these scales often have disadvantages; for example, they may fail to identify the root cause of a functional deficit, demonstrate a ceiling effect, are subject to clinician subjectivity or take too long to administer[3]. Examples of these different scales include the Berg Balance Scale, the Timed Up and Go (TUG) test, and the Balance Evaluation Systems Test (BESTest), each of which have one or more of the previously mentioned disadvantages to evaluating faller risk[4].

Identification of subtle musculoskeletal deficiencies in a clinical setting can be difficult, despite careful strength testing and the development of several strength scales. However, the subtle neuromuscular deficits that are associated with musculoskeletal pathology may be the most important predictors of disease progression, injury risk or treatment outcomes and help target rehabilitation strategies. It is often necessary to utilize more sophisticated tools such as dynamometry, motion analysis testing and intramuscular electromyography - which can be expensive and are not readily available in outpatient orthopaedic clinics.

Clinician-Friendly Assessment Tools (CATs) are inexpensive, easy-to-use, portable technological devices that can fill this gap by providing useful information for assessing musculoskeletal pathology. Currently, CATs are used as a part of rehabilitation exercises for previously injured patients, but are not routinely used for diagnosis or assessment[5]. The introduction of CATs into the physical exam portion of patient care could be crucial for preventing fragility fracture in a significant number of patients, especially those who have sustained a fragility fracture in the past. Previous studies have identified the following to be risk factors for fragility fracture: decreased muscle strength, core control, and increased postural sway, which is a good overall indicator of stability and balance in a given position [6-9]. CATs may offer an additional advantage to physical exam techniques by measuring these factors through detection of subtle differences in balance, coordination, movement and strength during functional movements such as walking, twisting and standing.

The purpose of this study is to examine the ability of CATs to predict fall risk; specifically, this study will evaluate how scores obtained by CATs correlate with scores from validated surveys which reliably predict fall risk. The results will be useful in determining the role of CATs in outpatient clinics as a part of fall risk screening. This also serves as the first of many studies to determine the reliability and accuracy of CATs.

## MATERIALS AND METHODS

### Recruitment

Institutional Review Board approval was obtained for this study prior to its initiation. Twenty patients who sustained fragility fractures over the age of 50 participated in this study. Hip fractures have long been associated with fall risk and osteoporosis, but recent research shows that fragility fractures at various other locations are associated with similar pre-existing comorbidities and increased morbidity after fracture[10]. Thus, patients sustaining fragility fractures of various locations were included in this study. Patients who had received surgery for a lower extremity fragility fracture were required to be at least two months out from their surgery to allow adequate recovery time; test group subjects were also required to be able to walk 40 feet unassisted. There was no maximum time since injury, since all patients with a history of fragility fracture are considered to be at risk for re-fracture. Twenty-one patients over the age of 50 without any history of fragility fracture were also recruited as a control group. Recruitment occurred at three different orthopaedic clinics. Relevant characteristics from each group are listed in Table 1. Distribution of fractures in the test group is listed in Table 2.

**Table 1:** Demographics

	Test	Control	*P*
N	20	21	0.34
Male	6	10	
Female	14	11	
Age (average)	64.0 ± 8.8	62.4 ± 8.9	0.57

### Equipment setup

**Figure 1.**
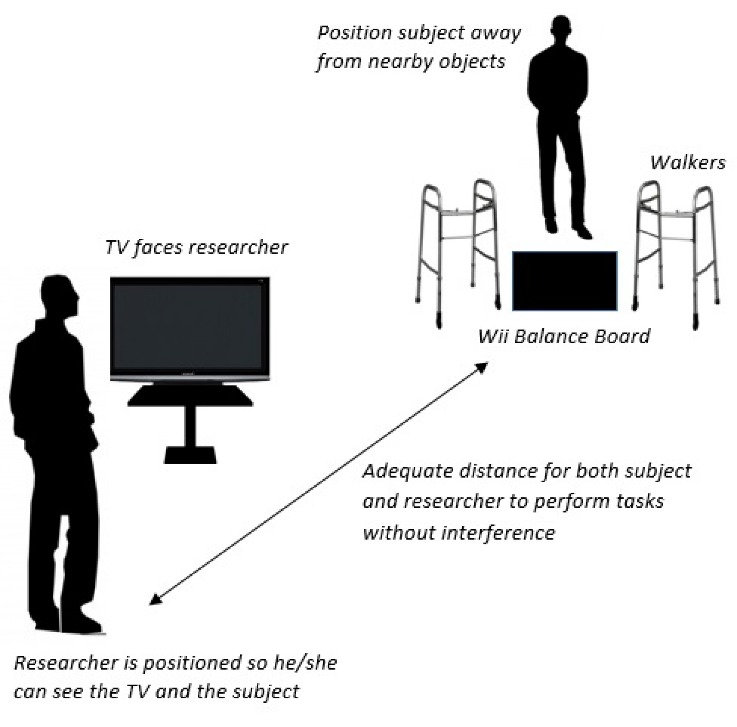
**Wii Balance Board setup**. The television is positioned away from the patient and the Balance Board is placed safely.

This study used three different technologies: the Wii Balance Board, iPod Level Belt Pro application, and Saehan hand grip dynamometer. The Wii Balance Board was placed an adequate distance away from any sharp objects and the research assistant, so that both the participant and research assistant could rotate with their arms at a 90 degree angle from their bodies while avoiding contact with any obstacles (Fig. 1). The television screen was only made visible to the research assistant to eliminate confusion, as some tasks selected by the research team were not consistent with the demonstrations provided by the Wii program. Standard folding walkers without wheels were placed on the right and left sides of the board to ensure patient safety, and patients were instructed to stabilize themselves using the walker whenever they felt unstable. Foldable walkers were selected to maintain portability and affordability.

The iPod Level Belt was placed directly superior to the subject’s posterior superior iliac spines, and secured anteriorly by Velcro. The only space required for this task was a hallway of at least 40 feet. The hand grip dynamometer required no extra space.

**Table 2:** Distribution of Fracture Location in Test Group

**Upper Extremitry**	8
**Lower Extremity**	9
**Both Upper + Lower**	3
**Total**	20

### Procedure

**Figure 2.**
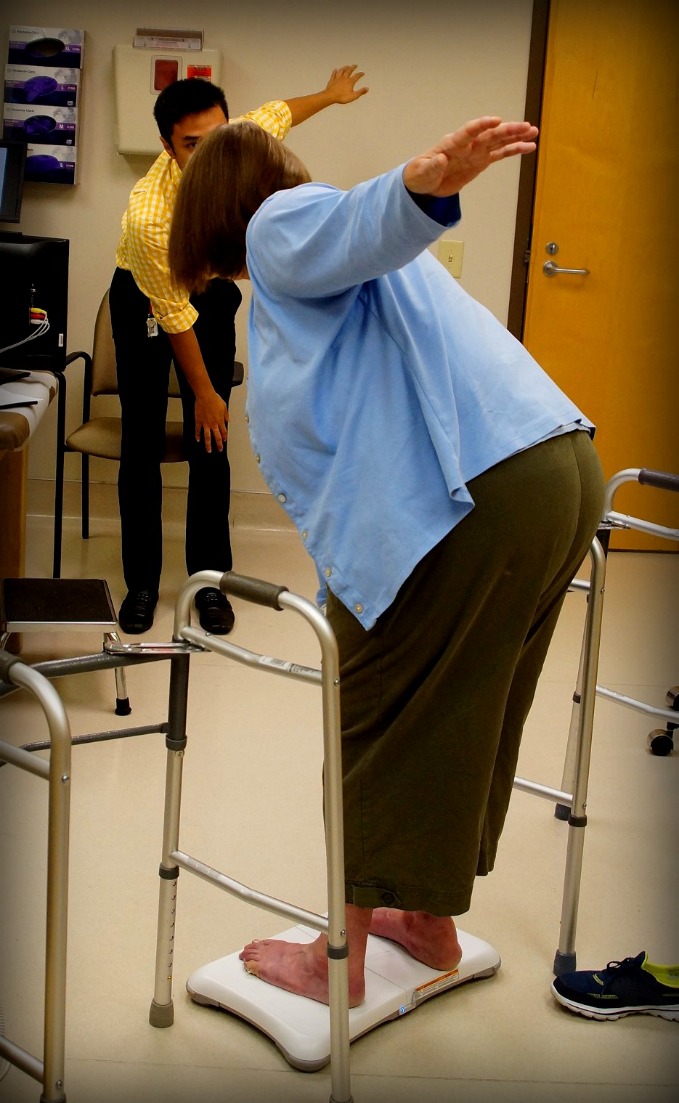
**Torso twists**. A subject performs Torso Twists on the Wii Balance Board, guided by a research assistant. Note that the subject’s shoes are removed to eliminate additional variables, and walkers are in place to ensure subject safety.

Each subject filled out an FES-I Fear of Falling survey, a validated survey that evaluates fall risk based on self-reported “fear of falling” during daily activities^2^. The test is a reliable, repeatable measure of concern surrounding activities that cover a range of difficulties and circumstances[8]. Each subject then filled out a patient demographics questionnaire that also served to screen ineligible patients: those with movement or sensory disorders such as Parkinson’s or peripheral neuropathy, those who were unable to walk 40 feet unassisted, and those that were less than two months out from a fragility fracture-related surgery. Included in the patient questionnaire was a section from the SF-36 Health Survey, which evaluates daily activity restrictions based on the subject’s current state of health. This portion of the survey states, “The following questions are about activities you might do during a typical day. Does your health now limit you in these activities? If so, how much?” The subject is then asked to determine if they are limited during ten different activities, such as “bathing or dressing yourself” or “walking one block”. Subjects have three options: “Yes, limited a lot,” “Yes, limited a little,” or “No, not limited at all.” Point values from 1 (limited a lot) to 3 (not limited at all) are assigned to the different answers to calculate each subject’s score out of a possible total of 30 points. This score is then divided by 10 (for the 10 different activities) to get an average activity score. Thus, subjects with higher scores are believed to have fewer activity restrictions on a daily basis than subjects with lower activity scores. The survey has been shown to be an accurate self-estimation of physical tasks[11]. Each subject was tested using the Nintendo Wii balance board, iPod Level Belt, and hand grip dynamometer. Each task was repeated three times, and the scores were averaged. Patients performed three trials of each test. Specific Nintendo Wii tests included Torso Twists, which evaluates double-leg stance postural sway during a medial-lateral sway task (Fig. 2), and a Single Leg Stand, which measures hip abduction strength, ankle stability, and postural sway. Both Wii Balance Board tasks were performed without shoes to eliminate additional variables caused by shoe type. During the Torso Twists task, subjects are asked to hold their arms and forearms parallel to the floor, with their feet hip distance apart. They were then instructed to twist 90 degrees in alternating directions from the waist and above. Subjects turned in each direction three times. For the Single Leg Stand, subjects were required to place their hands on their hips, but were allowed to balance with their lifted leg in whatever position was most comfortable, as we wanted these tests to be an accurate representation of functional everyday balance.

**Figure 3.**
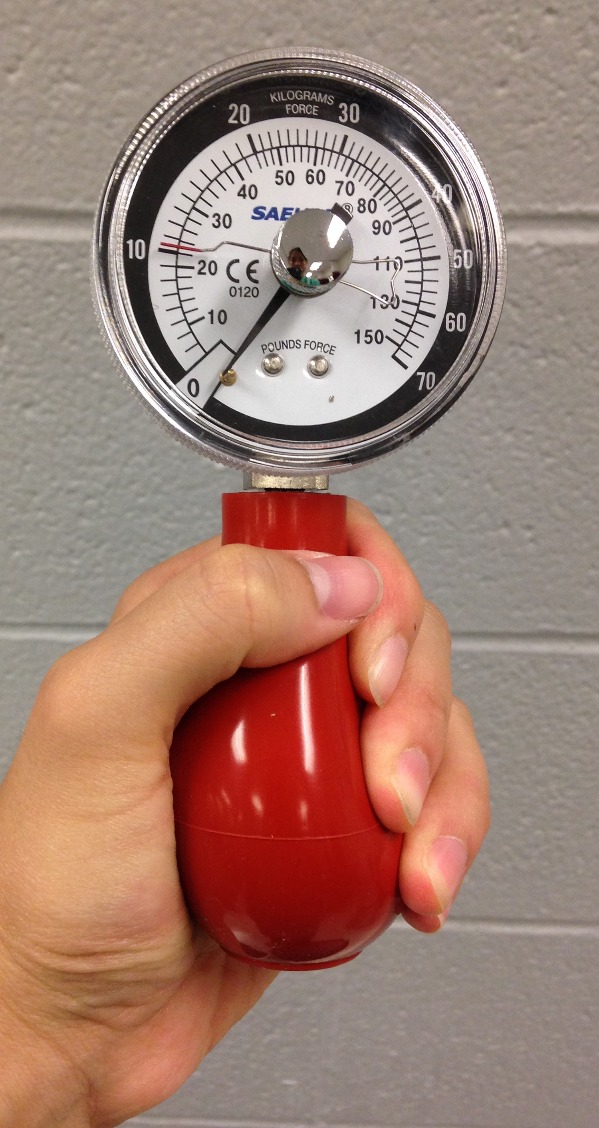
**Saehan hand grip dynamometer**. The subject is instructed to squeeze the red bulb, and grip strength is measured by the dynamometer.

Hand grip strength was then measured by asking patients to hold their forearm at a 90 degree angle to their arm with their elbow by their side, and squeezing the dynamometer balloon as tightly as possible (Fig. 3). This test was repeated with both hands. For this test, we recorded the maximum number achieved by each subject.

**Figure 4.**
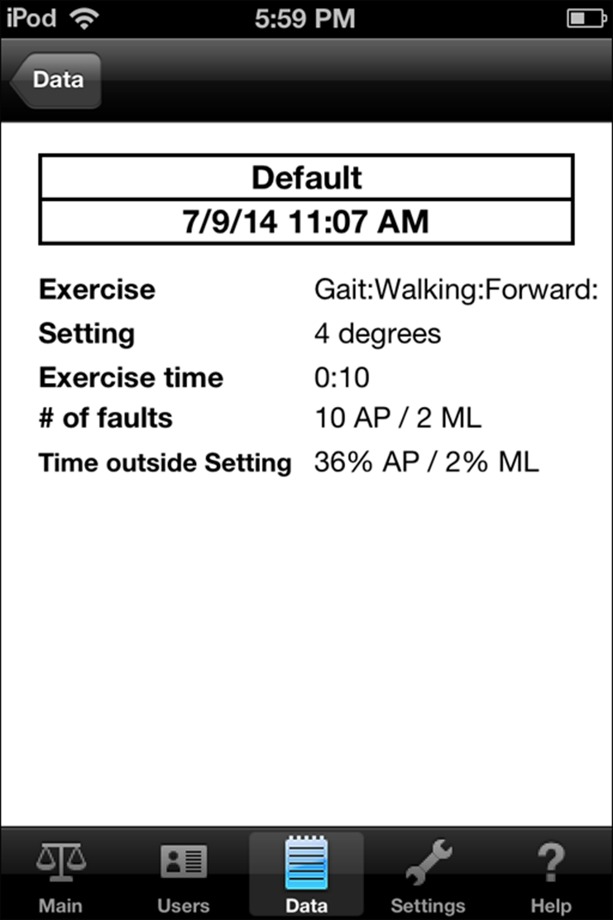
**Level Belt application**. iPod Level Belt application summary, as it appears on the iPod screen. Further data can be uploaded from this program to provide greater detail about a patient’s gait.

Subjects were then assessed during a daily, functional task: walking. Using the iPod Level Belt, subjects walked 40 feet to assess lumbo-pelvic (trunk) control, anterior/posterior and horizontal pelvic tilt, and balance[8]. Scores from the Wii Balance Board and hand grip dynamometer were displayed immediately. The degree of medial/lateral and anterior/posterior tilt is recorded at a rate of 60 readings per second by the iPod Level Belt application; an overall summary is also provided for each trial (Fig. 4). The time of completion for each trial was approximately 15 minutes.

### Analysis

A two-sample T-test was performed to investigate the differences between Fear of Falling (FES-I) scores and Activity level (SF-36) scores between test and control groups. Preliminary analyses on all scores were conducted to check for normality and outliers. Sensitivity analyses using a non-parametric procedure (Mann-Whitney) were conducted to confirm the robustness of each conclusion. The pairwise correlations between variables were summarized using Pearson correlations coefficients; Spearman correlation coefficients were used when the data was not normally distributed. The difference of correlation between test and control groups was investigated visually and tested using the linear regression where the test group was included as a covariate and as interaction term.

For the Nintendo Wii, the following variables were investigated: total Torso Twist scores (which included horizontal and vertical components), and total combined Single Leg Stance score for both legs. For the hand grip dynamometer, the maximum numbers achieved with the both hands were analyzed. With the iPod Level Belt, eight dependent variables were used: (1) average anterior-posterior sway, (2) maximum anterior-posterior sway, (3-4) average and maximum medial-lateral sway, (5) total anterior-posterior and (6) medial-lateral faults (instances that the subject exceeded the maximum movement range measured by the Level Belt), and average percentage of time out of (7) anterior-posterior or (8) medial-lateral range. These eight variables were used as different methods for measuring subjects’ core stability during walking, as increased postural sway and center of pressure displacement is shown to correspond to higher fall risk^[12]^. For the iPod Level Belt variables, only the middle 50% of the 40 m walk, when subject was walking at a constant velocity, was analyzed. The first and last 25% of each trial was eliminated to account for inconsistencies in subjects’ postures while the belt was being adjusted at the beginning of the trial, and how subjects decelerated as they reached the end of the hallway.

### RESULTS

The Fear score (FESI) was significantly higher for the test group (26.4±7.98) than the controls group (19.9 ± 3.84, p=0.0004). Average activity levels determined by the SF-36 survey were lower for the test group (2.11 ± 0.85) than for the control group (2.66 ± 0.42, p=0.014). Variables that showed a correlation with Fear of Falling scores were: maximum dominant hand grip strength, maximum non-dominant hand grip strength, average anterior-posterior sway measured by the iPod, and maximum anterior-posterior sway measured by the iPod. CATs scores demonstrating a significant correlation with FES-I scores are bolded and highlighted in green. While these tests are easy for providers to administer, and relatively easy for the able-bodied individual to complete, they can be intimidating for subjects who have little to no prior experience with the exercises, and who are recently recovering from injury. Out of respect for patient comfort, some tests could not be completed, which resulted in a sample size fewer than 40 for all tests. Thus, correlations with p values between 0.05 and 0.10 are considered to be moderately significant and are italicized and highlighted in yellow. These scores and their correlation coefficients are shown in Table 3. Four of the scores were shown to be at least marginally correlated (p=0.012 - 0.069) with FES-I values, while none of the scores obtained showed a significant correlation with SF-36 scores (Table 3).

### DISCUSSION

The purpose of this study was to determine if scores obtained via modern portable, inexpensive clinical assessment tools that assess function (such as balance) correlate with patient reported activity levels (via SF-36) and fear of falling (via FES-I). By measuring these correlations, we can better understand the implications of measurements obtained by CATs. The SF-36 and FES-I surveys were specifically selected because they have been shown to be reliable indicators of physical health and ability: activity levels measured by SF-36, and fear of falling measured by FES-I have been shown to consistently differ between those who have sustained musculoskeletal injuries and those who have not [11, 13]. Thus, the ability of CATs to record scores that correspond with FES-I and SF-36 scores is an important indicator of their clinical utility.

An evaluation of the clinical results reveals different ways in which CATs can assess the functionality. When the fracture group’s scores were closely examined, four scores (two from the iPod Level Belt, two from the hand grip dynamometer) were found to have a significant correlation with FES-I Fear of Falling scores. This suggests that CATs may have a role in providing a numerical measurement for the daily limitations that a fracture patient may face, such as walking, climbing stairs, taking a bath or shower, or getting out of a chair - all of which require a certain amount of strength, coordination and balance. It is possible that these limitations are due to physical restrictions, fear, or a combination of the two, and CATs are able to capture a complete picture. Numeric scores, provided by CATs, also provide a baseline from which providers could monitor progress throughout treatment and set goals that patients can easily understand.

**Table 3:** Activity (SF-36) and fear of falling (FES-I) scores.

Correlation with average activity (SF-36)				
**Tool**	**Variable**	**R**	**p**	**n**
**Wii BB**	Torso Twist - total	-0.197	0.230	39
	Single-leg stand - total	0.071	0.691	33
**Hand grip**	Maximum - dominant hand	0.162	0.330	38
	Maximum - nondominant hand	0.228	0.168	38
**iPod**	Average anterior-posterior SD	-0.183	0.284	36
	Average medial-lateral SD	-0.234	0.170	36
	Maximum anterior-posterior SD	-0.158	0.356	36
	Maximum medial-lateral SD	-0.209	0.221	36
**Correlation with fear of falling (FES-I)**				
**Wii BB**	Torso Twist -average	0.059	0.720	39
	Single-leg stand - average	-0.094	0.600	34
**Hand grip**	Maximum - dominant hand	*-0.302*	*0.069*	*37*
	Maximum - nondominant hand	*-0.309*	*0.059*	*38*
**iPod**	Average anterior-posterior SD	**0.416**	**0.012**	**36**
	Average medial-lateral SD	0.023	0.171	36
	Maximum anterior-posterior SD	*0.320*	*0.056*	*36*
	Maximum medial-lateral SD	0.270	0.112	36

Data in boldface are statistically significant (p < 0.05). Data in italic are marginally significant (p = 0.05 to to 0.10). SD = standard deviation.

One of the most common limitations among current clinical balance and strength scales is failure to identify the root cause of physical limitations among patients who receive low scores. Furthermore, once a patient receives a low score on one of these scales, implications for their treatment may still remain unclear. These CATs give providers a better idea of how subtle deficits in strength, balance and coordination affect their patients’ lives by capturing these deficits during everyday tasks, and also correlating these to FES-I and SF-36 scores. For example, the iPod Level Belt measures anterior/posterior and medial/lateral postural sway measured during a dynamic functional task (walking); values recorded by the Level Belt were shown to be significantly correlated with FES-I scores. While assessments such as TUG test may indicate numerous different pathologies leading to a poor score, CATs pinpoint specific deficits leading to a certain score and suggests its functional impact.

The tools used in this study provided valuable insight, but remained relatively inexpensive and portable. Altogether, the iPod with Level Belt attachment and hand grip dynamometer cost approximately $375. The setup was easily achievable by one person in 15 minutes. Between trials, the tools could be easily stored in a small area, so they do not require permanent space within the clinic.

Overall, this study demonstrates the utility of portable, inexpensive, commercially available tools such as the iPod Level Belt and handgrip dynamometer in a clinical orthopaedic setting. What is unique about these tools is their ability to capture data during functional tasks such as walking, twisting and balancing - giving providers a better idea of how a low score calculated with these tools affect patients’ daily movements. Scores obtained from the iPod Level Belt and handgrip dynamometer were shown to have a significant correlation with other measures of the effects of injury, specifically FES-I scores, which take both physical and emotional components of an injury into account. Looking forward, a prospective study with a greater number of participants would be the next step to test the ability of CATs to predict future falls with accuracy. Nintendo Wii, iPod Level Belt and hand grip strength testing takes less than fifteen minutes, and baseline scores could be easily and accurately obtained on a number of patients within a clinical setting. Furthermore, using these tools within a clinical setting requires minimal training and has been shown to be readily accepted by different members of the outpatient clinic staff [14].

Additionally, given that the CATs used in this study are commercially available and relatively inexpensive, patients could use these machines in their own homes to monitor progress as they complete cycles of physical therapy by recording scores on tests found to be reliable predictors of fall risk, such as walking with the iPod and testing grip strength. Previous studies have identified the benefits of Wii Balance Board training in patients with peripheral neuropathy[15], and continue to be of interest when evaluating the utility of different CATs tools in orthopaedic rehabilitation.

### Limitations

We have limited knowledge of how the subjects’ comorbidities and treatments affected their scores. While we required two months of healing time after a surgery to repair a fragility fracture, it is unclear if this amount of time was adequate to ensure that healing from the operation itself had a negligible impact on the score. Ideally, we would have data prior to and after fragility fracture for baseline comparison; however, current practices make it difficult to capture these patients prior to fragility fracture. Additionally, some of the subjects were hesitant to attempt the tests after just two months of recovery. This suggests that future studies should allow for a longer recovery period after injury, or that we need an additional “score” on our grading scale to account for those who are limited due to lack of comfort versus those limited due to lack of strength and mobility. In addition, our subjects had different comorbidities, including osteopenia, osteoporosis, joint replacements and carpal tunnel syndrome. When scores between subjects and controls are compared in the future to determine if CATs can differentiate these two groups, it will be important to match these subjects for type of joint replacements and other comorbidities, as it is unclear how these factors affect evaluation by CATs. The number of osteopenia/osteoporosis diagnoses between our two groups was the only comorbidity found to be statistically different between the two groups, with more diagnoses in the test group. However, this is likely due to increased testing for these conditions within fracture patients (only 3 of the controls had been tested, compared to 12 of the test subjects). Nevertheless, it is still important to evaluate how this comorbidity affects our testing, and strive to match our subjects in this area.

CATs tests captured a wide range of functional deficits in muscle strength, coordination and postural stability/balance which correlated with patient reported fear of falling and activities of daily living. However, this did lead to varying patient comfort with certain tasks, which impacted our sample size. This may be overcome in the future with a slightly more detailed introduction to the CATs tools to ensure patient comfort, as some patients’ unfamiliarity with the tasks may have impacted their decision to participate in certain parts of the testing.

### Conclusion

The results of this study identified the Saehan hand grip dynamometer and iPod Level Belt as two relatively inexpensive, portable tools that can identify subtle deficits in muscle strength, coordination, stability and balance. The hand grip dynamometer was used as an indicator for muscle strength that remained unaffected by lower extremity injuries. The iPod Level Belt showed the ability of CATs to capture the musculoskeletal deficits mentioned above during a common, daily movement - or “a dynamic functional task”: in this case, walking. These tools identify subtle deficits in gait that current physical exam techniques often miss, such as increased lateral or anterior-posterior sway, which may indicate decreased stability and predispose patients to falls; in addition, minor deficits in grip strength could be indicators of decreased strength and are not easily quantified on physical exam. Furthermore, CATs also give healthcare providers a numeric measure of patients’ limitations during daily activities due to their correlation with FES-I scores, so that providers do not have to rely on self-reporting. Targeting dynamic functional task deficits and determining how they affect patients’ daily activities is important for guiding rehabilitation and monitoring disease progression. In the future, CATs may also have a role in predicting outcomes and in further individualizing care, therapy, and at-home preventive measures.
